# From Observation to Information: Data-Driven Understanding of on Farm Yield Variation

**DOI:** 10.1371/journal.pone.0150015

**Published:** 2016-03-01

**Authors:** Daniel Jiménez, Hugo Dorado, James Cock, Steven D. Prager, Sylvain Delerce, Alexandre Grillon, Mercedes Andrade Bejarano, Hector Benavides, Andy Jarvis

**Affiliations:** 1 Decision and Policy Analysis (DAPA), International Center for Tropical Agriculture (CIAT), Cali, Colombia; 2 University of Applied Sciences of Western Switzerland (HEIG-VD), Yverdon-les-bains, Switzerland; 3 School of Statistics, Universidad del Valle, Cali, Colombia; 4 City government of Pereira, Secretary of rural development, project site-specific agriculture, Pereira, Colombia; Agricultural Research Service, UNITED STATES

## Abstract

Agriculture research uses “recommendation domains” to develop and transfer crop management practices adapted to specific contexts. The scale of recommendation domains is large when compared to individual production sites and often encompasses less environmental variation than farmers manage. Farmers constantly observe crop response to management practices at a field scale. These observations are of little use for other farms if the site and the weather are not described. The value of information obtained from farmers’ experiences and controlled experiments is enhanced when the circumstances under which it was generated are characterized within the conceptual framework of a recommendation domain, this latter defined by Non-Controllable Factors (NCFs). Controllable Factors (CFs) refer to those which farmers manage. Using a combination of expert guidance and a multi-stage analytic process, we evaluated the interplay of CFs and NCFs on plantain productivity in farmers’ fields. Data were obtained from multiple sources, including farmers. Experts identified candidate variables likely to influence yields. The influence of the candidate variables on yields was tested through conditional forests analysis. Factor analysis then clustered harvests produced under similar NCFs, into Homologous Events (HEs). The relationship between NCFs, CFs and productivity in intercropped plantain were analyzed with mixed models. Inclusion of HEs increased the explanatory power of models. Low median yields in monocropping coupled with the occasional high yields within most HEs indicated that most of these farmers were not using practices that exploited the yield potential of those HEs. Varieties grown by farmers were associated with particular HEs. This indicates that farmers do adapt their management to the particular conditions of their HEs. Our observations confirm that the definition of HEs as recommendation domains at a small-scale is valid, and that the effectiveness of distinct management practices for specific micro-recommendation domains can be identified with the methodologies developed.

## Introduction

Advances in agricultural technology are based almost entirely on observations made on crops, whether in the laboratory or the field. Over thousands of years, individual farmers have domesticated crops, selected improved cultivars [1] and learned how to manage them to suit the environment. Farmers’ knowledge and experience, combined with data from multiple sources is increasingly used as a tool for agricultural research and development [2–10].

Modern information technology complements farmers’ observations and adds an extra dimension to evaluation of agricultural system performance. Rigorous modern data mining techniques can establish relationships and associations between observations from multiple sources, which farmers can use to improve their crop husbandry [2–6].

The information flow of agricultural research and development is often a top-down process in which knowledge is generated at a few sites, normally laboratories or research stations, and is then passed on to farmers through a process of technology transfer. This process has provided remarkable improvements in productivity (see for example [7]). However, this top-down model frequently fails to take into account farmers’ knowledge, experience and needs and consequently may not be appropriate for their particular ambience [6,8–17]. The tools used successfully in this top-down model are also its Achilles heel; researchers reduce the confounding effects of temporal and spatial variation with controlled experiments in which a few factors are varied and the remainder are kept constant [11,18–21]. Experiments are designed to evaluate the effects of individual factors or combinations of a small number of factors for each crop within mega-environments or recommendation domains [22–25]. The recommendation domains are defined as “a group of farmers whose circumstances are similar enough so that they are all eligible for the same recommendation” [25]. This represents something of a disconnect between the research process and the agricultural reality. Farmers groups with similar conditions are the basis for the recommendations zones, whereas most of the research is directed towards mega-environments based on physical and biological characteristics that may cover a wide range of variation. The mega-environments do not take into account either the physical and biological features of a particular farm or the micro- environment or the idiosyncrasies of the farmers’ social space. Farmers’ social space includes such aspects as educational level, access to inputs, availability of labor, attitudes and all the other aspects that affect how they manage their farms.

Observations made by farmers will, either explicitly or tacitly, take into account not only their particular physical and biological environment, but also their social milieu. Hence, mega-environments and recommendation domains can be extremely successful across large areas with relatively homogeneous societies and ecologies, however they may not be appropriate when there is large degree of heterogeneity at a smaller scale within the mega-environment or recommendation domain. Nevertheless, conceptually recommendation domains evaluate options for crops grown under similar conditions, that is to say homogeneous growing conditions [25]. If it were possible to adequately characterize the conditions under which individual experiences occurred and observed, then the conclusions drawn from these observations and experiences could be transferred to sites that have similar conditions [26–29].

Historically, lack of information on the circumstances under which the farmers made their observations hampered their use in agricultural research. Precise characterization of growing conditions and management makes it possible to put both researchers’ and farmers’ experiences into context. The value of information and knowledge generated both from farmers’ experiences and controlled experiments is greatly enhanced when the circumstances under which it was generated are characterized. Early quantitative analyses in agriculture benefitted from operations research [30]. Towards the end of the twentieth century, precision agriculture began to be used as management strategies that use information technologies to bring data from multiple sources to bear on decisions associated with crop management [31]. Precision agriculture couples farmers' observations with precise characterization of the production conditions. This combination addresses the historical challenge of describing the context and provides insights into the crop response to multiple variables in a specific context, at both the micro-level within a field and between fields or management units [3].

Precision agriculture emphasizes within field variation, whereas site-specific agriculture, as we define it, looks principally at variation between sites or management units. A management unit is a set of plots or fields with relatively homogeneous environmental conditions and reasonably uniform agricultural management practices. Several studies suggest that, in the tropics at a site-specific level, one needs to steward the field or management unit well before attempting to adjust practices to handle the micro-spatial variation within a field [3,31, 32,33].

Data obtained from observations at the management unit level tend to be noisy with many factors that interact influencing the response of the dependent variables. To make sense of the variation large datasets and “Big Data” methodologies are needed [34–36].

To simplify analysis, we define two major classes of variables: Non-Controllable Factors (NCFs) which broadly define or characterize the recommendation domain; and Controllable Factors (CFs) which the farmer can manage to achieve his goals. Farmers can manage or influence the controllable factors (CFs), but learn to live with the NCFs.

The purpose of this paper is to illustrate how farmers`knowledge can be put into the context of recommendation domains, and hence determine the effects of CFs within a specific well defined set of environmental conditions or NCFs. This process enhances the value of farmers' observations obtained from an applied agricultural setting, so that the observations and experiences of the farmers provide them with an improved knowledge base from which they can make decisions relevant to their specific circumstances. Key features of this approach are: (a) the use of recommendation domains to explain part of the variance; and (b) a large number of observations so as to estimate stochastic variation within domains and reveal true associations of yield with management factors.

We bring together farmers’ observations and data from multiple sources for plantain (*Musa balbisiana*), an under-researched crop grown over a wide range of conditions in Colombia [37], to develop and test analytic approaches that increase our understanding of a crop to variations in CFs within the framework of NCFs or recommendation domains. The insights gained allow farmers to make better well informed management decisions.

## Materials and Methods

The approach integrates distinct types of observations on NCFs, CFs and crop response with expert opinions of both experienced agronomists and agricultural scientists, to provide a quantitative evaluation of the potential to manage CFs so as to increase productivity within the constraints of NCFs.

### Identifying data and selecting variables

Project variables were identified using a two stage process. An initial set of variables was determined through consultation with experienced agronomists and extension agents working with plantain. They identified a list of variables likely to influence crop productivity and quality. This list of variables was then compared with those in the available databases. When variables on the list were not readily available in existing resources, we systematically evaluated feasibility for inclusion. When feasible, tools were developed to collect data for the identified variables. When it was determined that either the costs outweighed the benefits or, simply, the data were unavailable as was the case with disease, pest, weed incidence and fertilizer use, the variables were discarded.

Soil characteristics were identified as important, but most growers neither have access to reliable information, nor have the habit of sharing the data, nor do they have a standard format for reporting soil traits. We used the well-established in situ methodology, RASTA (Rapid Soil and Terrain Assessment, available online at http://www.open-aeps.org/RASTA.pdf), to characterize soils [38].

The variables plant density, intercropping, variety, planting pattern, and annual production were identified by both experienced agronomists and agricultural scientists. Using both internet-based and facilitated paper-based survey instruments, farmers were asked to recall the practices used over the past year. Geographical coordinates were obtained by Global Positioning System (GPS) or from Google maps. Because farmers were being asked to reconstruct the characteristics of the growing season from memory, it was unlikely that we would be able to obtain reliable data on pest and disease management and fertilization. Hence, this data was not requested. The data was collected over a period of approximately two years during 2011 and 2012. Collected data were then standardized, and a single database of production events was established.

The standardized database registered 1322 cropping events. Of these, 1055 were obtained through the facilitators employed as part of the project and 267 were registered on-line by the farmers themselves. In this dataset there were adequate data to characterize 998 cropping events. Three hundred and twenty four (324) records were removed from the database due to inconsistencies including obvious errors in GPS. The geographic locations of the 998 geo-referenced cropping events were linked to climate data from the WorldClim database (http://www.worldclim.org/bioclim) [39]. This database is an interpolated climate surface for global land areas at a spatial resolution of 30 arc seconds (1-km spatial resolution). WorldClim is publicly-available and contains data on monthly precipitation and mean, minimum and maximum air temperatures. We extracted 19 WorldClim bioclimatic variables for each event. Soil and terrain characteristics of 752 cropping events were determined with the RASTA methodology. The climate, soil and crop management data acquired are summarized in Table 1.

**Table 1:** Types, levels of measurement and sources of collected observations.

Inputs	Type	Scale	Acronym	Source
**Controllable Factors (CFs)**				
Plant density	Quant^b^	Ratio	Pl_dens	ProjectDB
Cropping systems (monocropping, intercroppping)	Qual^a^	Nominal	Int	ProjectDB
Variety	Qual^a^	Nominal	Var	ProjectDB
Planting pattern	Qual^a^	Nominal	Pl_patt	ProjectDB
**Non-Controllable Factors (NCFs)**				
**Soil**				
Texture	Qual^a^	Nominal	Text	RASTA
Effective soil depth	Quant^b^	Ratio	Eff_depth	RASTA
Soil organic matter	Qual^b^	Ordinal	SOM	RASTA
Drainage	Qual^b^	Ordinal	Drain	RASTA
pH	Quant^b^	Interval	pH	RASTA
**Climate**				
Annual Mean Temperature	Quant^b^	Interval	BIO_1_	BIOCLIM
Mean Diurnal Range	Quant^b^	Ratio	BIO_2_	BIOCLIM
Isothermality	Quant^b^	Ratio	BIO_3_	BIOCLIM
Temperature Seasonality	Quant^b^	Interval	BIO_4_	BIOCLIM
Max Temperature of Warmest Month	Quant^b^	Interval	BIO_5_	BIOCLIM
Min Temperature of Coldest Month	Quant^b^	Interval	BIO_6_	BIOCLIM
Temperature Annual Range	Quant^b^	Ratio	BIO_7_	BIOCLIM
Mean Temperature of Wettest Quarter	Quant^b^	Interval	BIO_8_	BIOCLIM
Mean Temperature of Driest Quarter	Quant^b^	Interval	BIO_9_	BIOCLIM
Mean Temperature of Warmest Quarter	Quant^b^	Interval	BIO_10_	BIOCLIM
Mean Temperature of Coldest Quarter	Quant^b^	Interval	BIO_11_	BIOCLIM
Annual Precipitation	Quant^b^	Ratio	BIO_12_	BIOCLIM
Precipitation of Wettest Month	Quant^b^	Ratio	BIO_13_	BIOCLIM
Precipitation of Driest Month	Quant^b^	Ratio	BIO_14_	BIOCLIM
Precipitation Seasonality	Quant^b^	Ratio	BIO_15_	BIOCLIM
Precipitation of Wettest Quarter	Quant^b^	Ratio	BIO_16_	BIOCLIM
Precipitation of Driest Quarter	Quant^b^	Ratio	BIO_17_	BIOCLIM
Precipitation of Warmest Quarter	Quant^b^	Ratio	BIO_18_	BIOCLIM
Precipitation of Coldest Quarter	Quant^b^	Ratio	BIO_19_	BIOCLIM
**Geographical Coordinates**	Quant^b^	Interval	GPS	ProjectDB
**Output**				
Plantain yield	Quant^b^	Ratio	Yield	ProjectDB

^a^ Categorical variables.

^b^ Continuous variables

More than a half of the observations corresponded to intercropped plantain. As the productivity measurements from the two different systems were ton ha^-1^ year^-1^ for monocropping and kg plant^-1^ year^-1^ for intercropping, the dataset was split into two new datasets (monocropping and intercropping) for analysis. All other measurements were standardized and obvious outliers and errors eliminated.

### Conditional forest analysis of overall dataset

A major advantage of a standardized database is that all the information required for the analysis is contained within a single dataset. A challenge, however, with analyzing such datasets is the “small n large P” conundrum wherein the number of predictors relative to the total number of observations may limit traditional degrees of freedom. Likewise, the mix of categorical and continuous variables, variable quality of the individual observations, missing values and inter-predictor correlations suggested that a flexible analytic approach, such as those found within the family of random forest techniques, would be appropriate [40,41].

Categorical variables tend to introduce bias into the tree selection process in typical random forest models. A variation of random forests, conditional forest, was used to overcome the bias associated with variable selection in individual classification of trees, through a conditional inference framework with the goal of minimizing the influence of categorical variables and numbers of categories on the more typical split criterion [40]. The resultant conditional variable importance measure [41] evaluates both the marginal importance (Does this variable help to explain the response?) and the conditional importance (Does this variable help to explain the problem better if all the other variables are already given?) of each variable described in the observation set. While conditional forest models may be used to develop predictive models, we used the results of the conditional forest to choose the most appropriate clustering techniques. In the present study, analyses were conducted using the package “part(y)itioning laboratory assembling various high- and low-level tools for building tree-based regression and classification models”, also known as “party” in R (www.r-project.org). We initially analyzed the overall dataset with a conditional forest model to determine which CFs and NCFs were associated with productivity and their relative importance. The results of this initial analysis were then compared with the sets of variables suggested by agronomists and agricultural scientists. The conditional forest methodology was then applied to various combinations of factors. The methodology required complete datasets with yield and all NCFs and CFs. As the datasets were often incomplete, the yield datasets analyzed were reduced to 467 from the initial 998 cropping events.

### Clustering homologous events

Previous studies suggested that the most effective means of analyzing data obtained from multiple sources, including field observations, is as follows: first, clustering production within homologous groups for NCFs, and secondly, looking for yield variation within each cluster [3,26,28,42]. The homologous groups, denoted as Homologous Events (HEs), occur, in both space and time.

In order to cluster environmental information, and to incorporate as many of the previously identified explanatory variables as possible, we used factorial analysis for data reduction and hierarchical clustering based on Ward’s method, with K-means for data partitioning [43–47]. In the case of climate we chose Principal Components Analysis (PCA). This technique was chosen given its ability to: a) include continuous variables; b) retain the principal components; and c) minimize the effects of the high degree of linear correlation of the variables included in the WorldClim database. In addition, its combination with Ward’s method and K-means generated a defined number of clusters [48,49]. The method consists in first PCA to reduce the dimensionality of the original data, second, hierarchical clustering based on the principal components suggested by the PCA analysis, in such a way that the minimum within-variance in each cluster is retained, and third a partition of clusters by the K-means algorithm [49]. Applications of these techniques in agriculture and clustering climate data can be found in [47,50–56]. In the present study we implemented a package for exploratory data analysis in R called FactoMineR (http://factominer.free.fr), to cluster the climate data. For soils, we followed the same procedures, but instead of using PCA as a factorial analysis for data reduction, we employed Categorical Principal Components Analysis (CATPCA). The method was selected given that the soil dataset included both quantitative and qualitative information presented in nominal, ordinal, and continuous scale (Table 1). CATPCA is a factorial analysis well-suited to treat these types of variables of different scales; it converts every category to a numeric value through a process called optimal quantification (optimal scaling/scoring) [57]. Several studies have selected CATPCA as the most apposite factorial analysis to deal with quantitative and qualitative variables present in their datasets [58–61]. In this paper CATPCA was executed in the categories module of SPSS/ PASW 18^®^ [62].

### Understanding linkages between non-Controllable factors, controllable factors and productivity

Various models were considered for understanding the relationships between NCFs, CFs and productivity. Mixed models combined with Generalized Least Squares (GLS) were chosen given their ability to: (a) provide a quantitative estimate of the effects of CFs on yield within a hierarchical framework of clusters of HE; (b) estimate the dependencies between the independent and dependent variables; (c) keep the initial units of the dependent variable, which in our case is fruit yield; and (d) handle datasets with both continuous and categorical variables. These techniques were chosen and used on the same pragmatic basis followed by several researchers [26,28,63–66]. The method has been be used to compare the performance of varieties grown under a range of conditions in commercial fields with all varieties not being grown at all sites [63,64,67]. In our datasets, most of the varieties were not grown in all HEs. In the present study we used the linear and Non-Linear Mixed Effects models (NLME) package in R to fit a linear mixed model for the prediction of random effects (the term prediction is normally used for the estimation of random effects, whereas estimation is used for fixed effects) [63,64].

## Results

In this section we address the information gained in each stage of the multi-stage analytical process. The data are summarized and some of the fundamental characteristics that differentiate plantain cropping systems described. Based on these differences the data were pooled in order to understand the potential influence of management practices, soils and climate on yields. We contextualized management practices within sets of similar domains that emerge from the various combinations of NCFs that are grouped in HEs. Finally, the interactions between management practices or CFs and HEs were identified.

A simple first look at the data showed a skewed distribution of yield for both monocropping and intercropping systems, with a small number of management units with high yields and most of the management units with low yields (S1 Fig).

### Analysis of overall dataset using conditional forest

**Fig 1:**
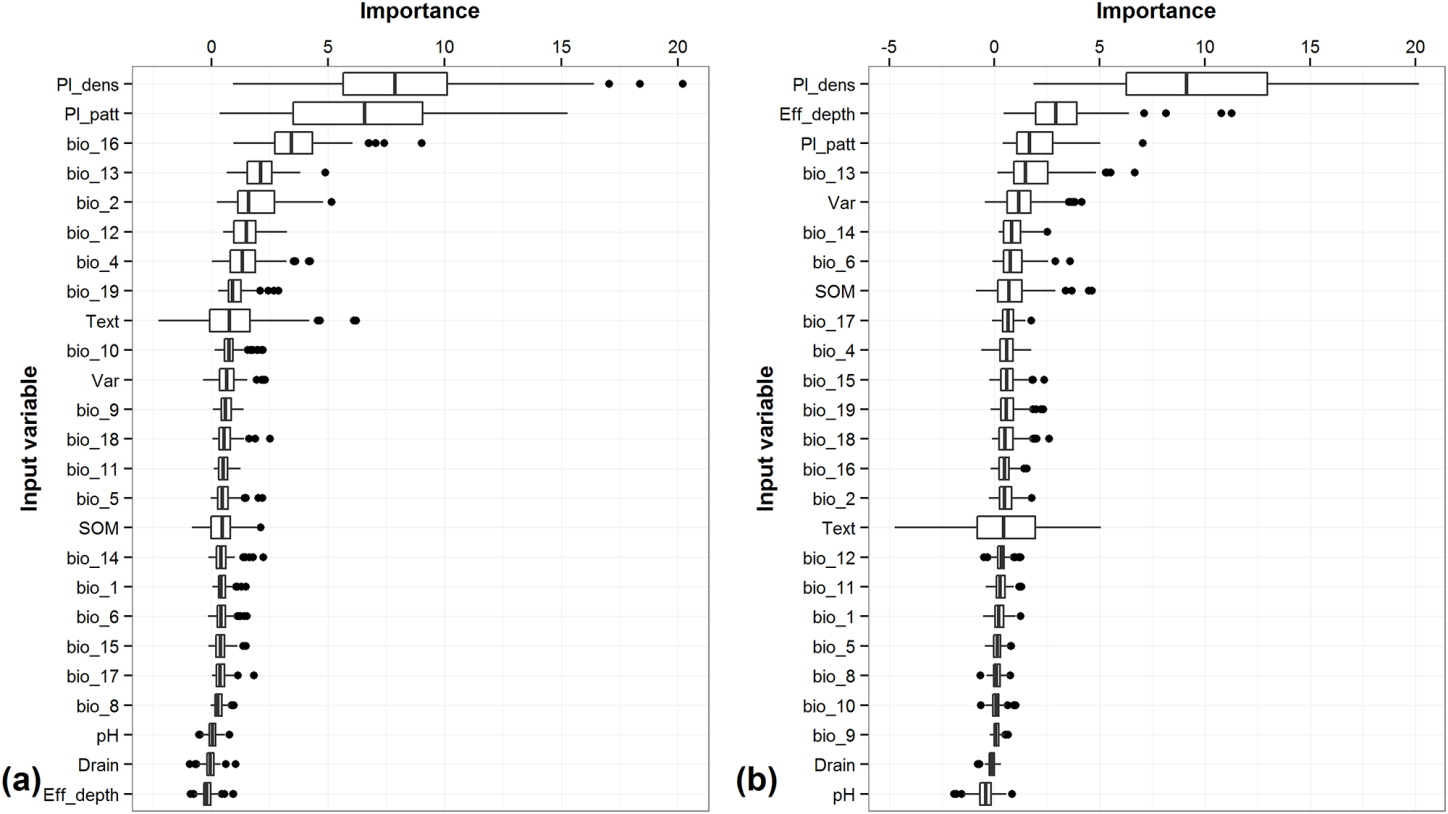
Variable importance for both systems. (a) monocropping, (b) intercropping.

The relative importance of each of the identified variables was computed with the conditional inference trees for each of the two systems. In monocropping systems, the conditional variable importance measure indicates that management practices, specifically plant density and planting pattern (Fig 1), both controllable factors, have the greatest influence on yield. The remaining top ten variables with the highest variable importance are all NCFs, of which one is soil texture and the others are related to climate.

In the intercropping system, plant density and planting pattern were again highly explanatory, as the first and third most important variables. In contrast to the monocropping system, an additional CF, plant variety, also influenced yield (Fig 1). Interestingly, the top ten variables in the intercropping system included two variables characterizing soils, including effective soil depth and soil organic matter, neither of which were as relatively important in the monocropping system. Of the climate variables, both systems indicated a combination of temperature and precipitation variables in the top ten (Fig 1). The conditional forest demonstrated that many of the factors identified by the experts were indeed important determinants of yield, or at least were associated with variation in yield. The inclusion of combinations of factors in the conditional forest analysis did not provide further insights into those combinations of factors that most affected yield.

### Clustering homologous events. (understanding linkages between non-controllable factors, controllable factors and productivity)

The management units were clustered into HEs using data from both the intercropping and monocropping datasets. We first clustered environmental information, through the combination of factorial analysis for data reduction and hierarchical cluster (S2 and S3 Figs). In the case of climate, four components with eigenvalues greater than 1 explained 92.1% of the total variance. For soils, the first two components explained 61.6% (Table 2).

**Table 2:** Eigenvalues for the principal components in both climate and soil datasets, and the variables with more contribution to each climatic and soil component.

Component	Eigenvalue	Cumulative % of variance	Characteristics
			**Climate**
**1**	8.49	44.71	Mainly contribution of temperatures (BIO_1_, BIO_5_, BIO_6_, BIO_8_, BIO_9_, BIO_10_, BIO_11_)
**2**	5.25	72.36	Highly associated with precipitation (BIO_12_, BIO_13_, BIO_16_, BIO_17_, BIO_18_, BIO_19_)
**3**	2.49	85.44	Mainly contribution of temperature variability (BIO_2_, BIO_3_, BIO_4_, BIO_7_)
**4**	1.28	92.17	One component in mean diurnal range (BIO_2_) and the other in precipitation seasonality (BIO_15_)
			**Soil**
**1**	2.26	37.69	Mainly contribution of IntDrain and SOM
**2**	1.43	61.59	Highly associated with ExtDrain and texture

When applying the Ward`s technique to the data, the mathematical procedure assessed from 2 to 15 clusters. The technique consistently suggested 2 clusters for climate and 3 for soils. Nevertheless, the range for some variables was wider than that considered to be suitable for a homologous event for plantain: thus, for example, the temperature range within clusters was felt to be too large. Experts suggested that up to about 15 clusters or recommendation domains was a reasonable, subjective assessment for the range of conditions in our study. Therefore, the clustering procedure was configured to evaluate 5 to 15 clusters. The clustering procedure indicated 7 climate clusters and 7 soil clusters within this new framework (S2 and S3 Figs). On inspection by the experts, the ranges within individual clusters appeared acceptable. Hence, these revised clusters were used as input for the K-means partition [46,49].

**Fig 2:**
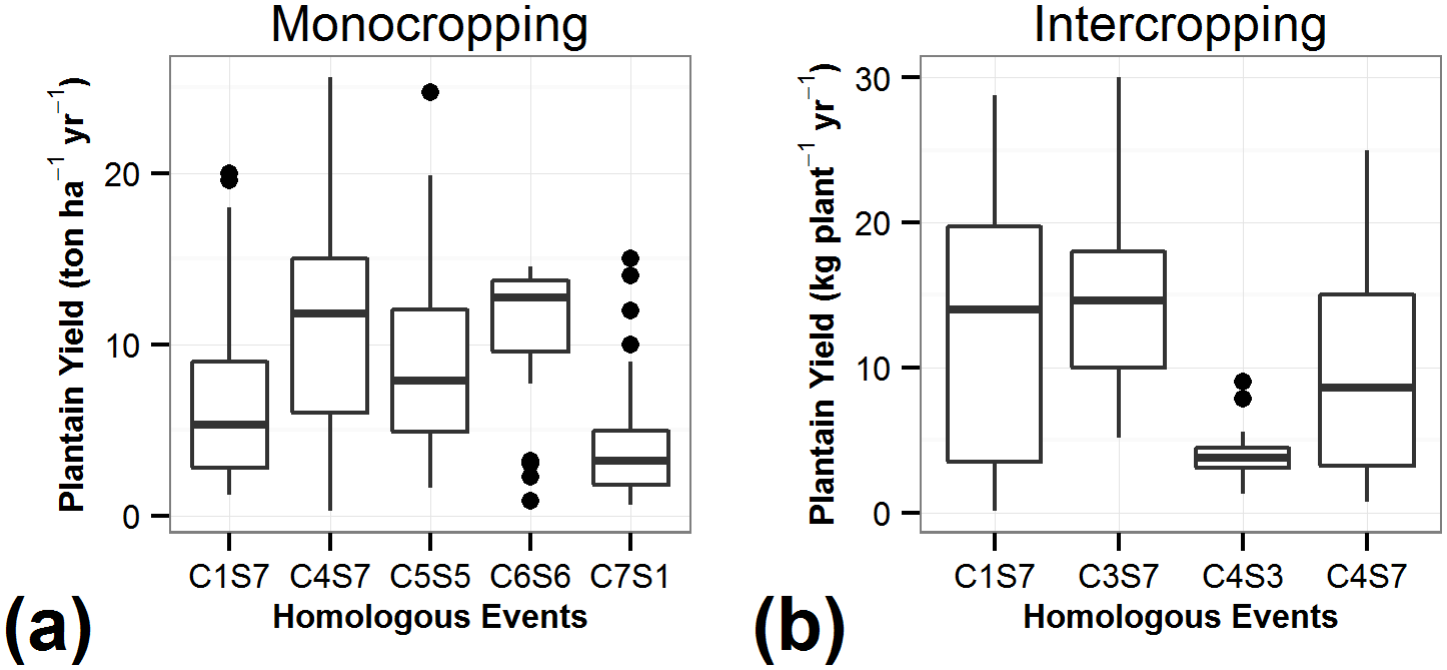
Yield distribution across the HEs for both systems. (a) monocropping, (b) intercropping. In both figures, C indicates climate whereas S specifies soils. The combination of CS designates each HE considering both environmental factors.

Differences between crop performance grown under identical environmental conditions (NCFs) can be attributed to crop management (CFs) [3,68–70]. Theoretically, as the variance in NCFs within an HE decreases, the proportion of the variance explained by CFs or management increases. Assuming that the HEs are relatively homogeneous, with minimal variance for NCFs that influence crop performance, most of the variance in productivity would be related to variation in the management practices or CFs. Plantain yield was assessed within each HE. Yield varied greatly within HEs (Fig 2). The highest yield in the HE C3S7 intercropping system produced 30 kg plant^-1^ yr^-1^ whereas the lowest was 1.67 kg plant^-1^ yr^-1^. In monocropping in HE C4S7 the highest yield was 29 ton ha^-1^ yr^-1^ whereas the lowest was 0.3 ton ha^-1^ yr^-1^. This indicated that HEs explained part of the yield variance, but that controllable factors (CFs) also had a major impact on yield within the HEs.

In order to analyze the relationships between NCFs, CFs and productivity, we chose mixed models to handle both fixed and random effects [71] and also missing values [63,72].

### Intercropping

We initially structured the model with HEs as a random effect with fixed effects of variety, plant density and planting pattern. For intercropping, the method suggested a structure including: (a) HEs as a relevant variable associated with yield and (b) different performance of varieties across HEs. Fixed effects for this model included the CFs: plant density and planting pattern. (Table 3).

**Table 3:** Frequency table Homologous Events versus Variety and Planting pattern for both monocropping and intercropping.

	Monocropping (HEs)					Intercropping (HEs)			
Variety	C1S7^a^	C4S7^a^	C5S5^a^	C6S6^a^	C7S1^a^	C1S7^a^	C3S7^a^	C4S3^a^	C4S7^a^
Dominico						1	14	0	0
Dominico-harton	6	24	0	0	0	70	13	30	36
Harton	15	0	51	33	50	13	4	0	0
Mixture						16	20	0	2
Other						0	31	0	0
***p*-value**^b^					< 0.0				<0.0
**Planting pattern**									
Square	19	20	2	5	50	28	82	30	30
Quincunx	4	4	49	29	0	72	0	0	8
***p*-value**^b^					< 0.0				< 0.0

^a^ Homologous Events. C indicates climate, whereas S specifies soils, Combination of CS designates each homologous events considering both environmental factors

^b^ *p*-values were calculated using the Fisher's exact test for count data

Inspection of the data indicated that management practices were closely associated with HEs. For example in intercropping, the planting pattern was closely linked with the HE. The Fisher test indicated that HEs and planting pattern were not independent (*p* = <0.05) (Table 3). Consequently, in a complete analysis of the overall dataset with mixed models, HEs/NCFs and CFs, would be confounded. Hence, effects of CFs on yield were also evaluated within HEs. The model explaining yield with the lowest Akaike Information Criterion was:

$$Yield_{ij}=11.20+5.48PQuin-0.0038Dens+\alpha_{j}+b_{j1}\text{Dominico}+b_{j2}\text{Harton}+\;b_{j3}\text{Mixture}+\;b_{j4}\text{Other}+\;\varepsilon_{ij}$$ (1)

$$\varepsilon_{ij}\sim \;N\left(0,\sigma^{2}\right)\;\;\;\;\;\;\;\alpha_{j}\sim \;N\left(0,\sigma_{a}^{2}\right)\;\;\;\;\;\;\;b_{jk}\sim \;N\left(0,\sigma_{bk}^{2}\right)$$

Where *Yield* = Yield kg plant^-1^ yr^-1^, *PQuin* = planting pattern Quincux with square planting as reference, *Dens* = Density (plants/ ha), *α*_*j*_ = The additional change in yield due to HE*j* (*j* = 1,2,3,4) and *b*_jk_ = the additional change in yield due to the variety *k* instead of Dominico-harton for HE *j* (*k* = 1,2,3,4).

The coefficient *β*_1_ = 5.48 suggested yield with the quincunx planting pattern was 5.48 kg plant^-1^ yr^-1^ greater than with square planting. The effect was consistent in those HEs where both square planting and quincunx were used and could be compared (S4 Fig). However, the agronomists and extension agents on the team did not believe that changing from square planting to quincunx alone would produce this large yield difference. They observed that the farmers who used the quincunx system tended to be more progressive: The quincunx system was part of a superior technology package, but was the only measured parameter indicative of that package.

To test this hypothesis, particular regions were used as proxies for technology levels (technology proxies) on the tenuous assumption that farmers in the same region interchange ideas and tend to use the same technology packages. We divided the growers into groups from the Central Coffee growing region (commonly known as *the eje cafetero* in Colombia), the Southern Andean region (Cauca and Nariño departments which tend to be poorer and less well developed), and the Inter-Andean regions of Valle, Tolima and Santander which extension agents indicated used a more intermediate technology. With a mixed model the restricted maximum likelihood using region instead of planting pattern was given by:

$$Yield_{ij}=16.56+4.21TecnCoffee-5.00TecnSouthAnd-0.0042Dens+\alpha_{j}+b_{j1}\text{Dominico}+b_{j2}\text{Harton}+\;b_{j3}\text{Mixture}+\;b_{j4}\text{Other}+\;\varepsilon_{ij}$$ (2)

In this function, *TecnCoffee* (technology from coffee region) was the proxy for the Central Coffee growing region, *TecnSoutAnd* (technology from Cauca and Nariño departments) was the Southern Andean region with the *InterAndean* (technology from inter-Andean region) taken as the standard for comparison.

The *TecnCoffee* proxy yielded 4.2 kg plant^-1^ yr^-1^ more than the intermediate technology Inter-Andean zone and the Southern Andean technology produced 5.0 kg plant^-1^ yr^-1^ less. In both cases, whether using quincunx or the technology proxy, preproduction per plant decreased as plant density increased. The distinct varieties were concentrated in particular HEs, and the variety effect was likely confounded with HEs (Table 3).

**Fig 3:**
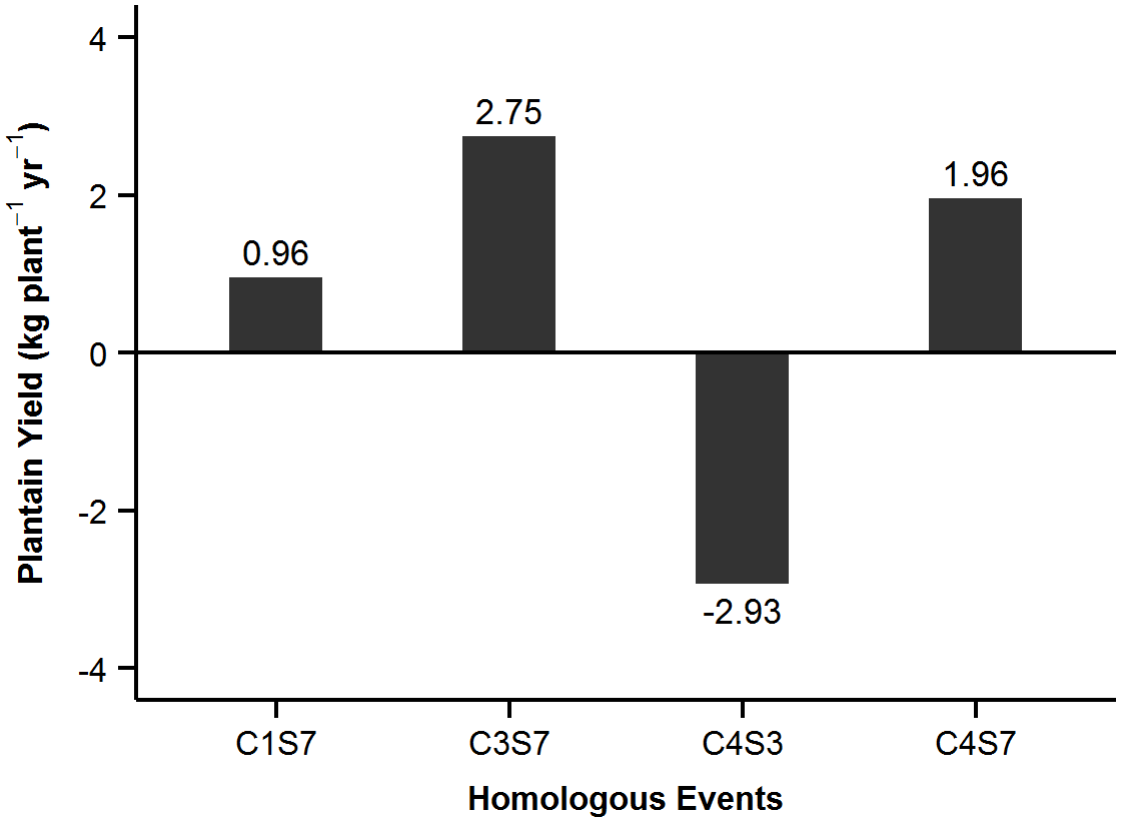
Effects of HEs in the mixed model for intercropping.

**Fig 4:**
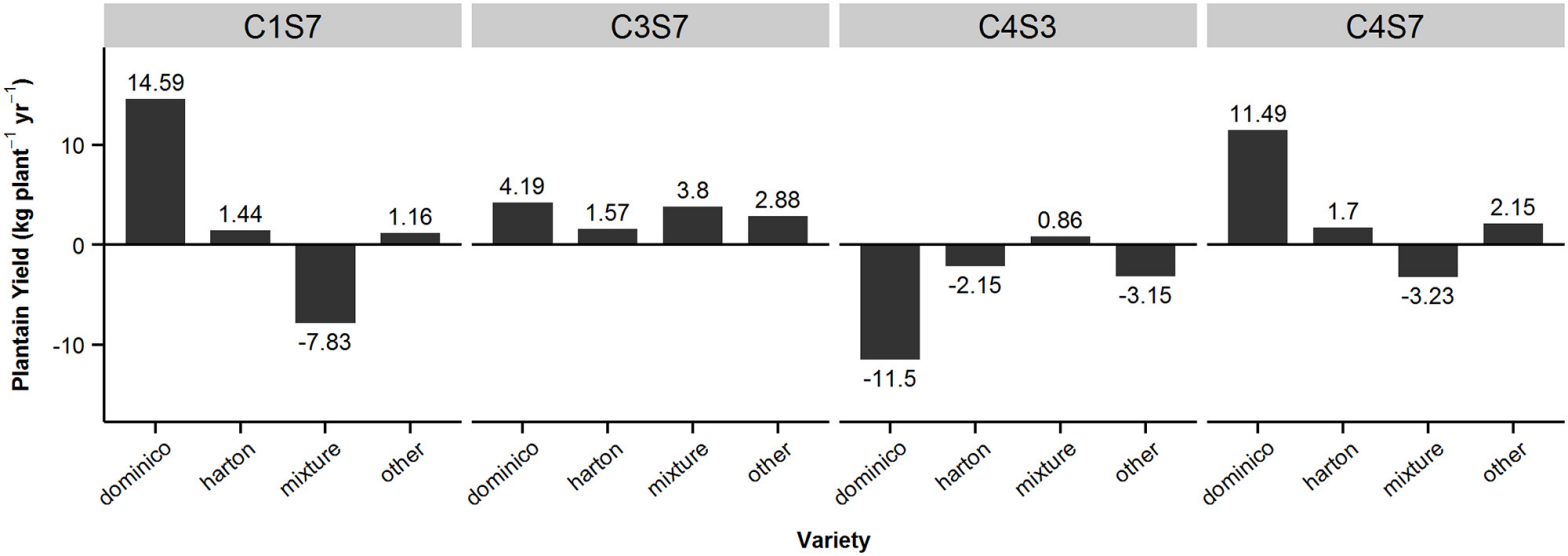
Effects of varieties across HEs for intercropping.

With the intercropping system, yields in C3S7 were 2.75 kg plant^-1^ yr^-1^ more than the average, whereas in C4S7 and C1S7 yields were 1.96 and 0.96 kg plant^-1^ yr^-1^ more. C4S3 was the least productive HE with 2.93 kg plant^-1^ yr^-1^ less than the average value (Fig 3). The mixed model estimated the yield of varieties in distinct HEs (Fig 4). The estimates suggest that: (a) in C3S7, Harton is the lowest yielding variety; (b) in C1S7 and C4S7, Dominico yields most; and (c) in C4S3, HE with the lowest yields, Dominico yields 11.5 kg plant^-1^ yr^-1^ less than the average value. However, the estimates of yield in the distinct HEs are largely based on the random effects of the mixed model. In C4S3, there was no data on Dominico. Thus, care is required when interpreting the information provided by mixed models in unbalanced datasets (Fig 4).

### Monocropping

The variation in yield was partially explained by the CFs with a significant effect of HEs on yield. The yield variation was least in C6S6 with a coefficient of variation (CV) of 34% (Table 4). The greatest yield variation within HEs was in C1S7 (CV 83%) and C7S1 (CV 82%). The yield variation in C6S6 was highly skewed towards high yield with a median yield of 12.7 ton ha^-1^ yr^-1^ and skewness coefficient of -1.55 (Table 4, and S5 Fig). On the other hand, the yield variation of C1S7 was highly skewed towards lower yield with a skewness coefficient of 1.12 and a median yield of 5.3 ton ha^-1^ yr^-1^. Similarly C7S1 had a skewness coefficient of 1.86 and a median yield of 3.2 ton ha^-1^ yr^-1^. The HEs of C4S7 (skewness coefficient 0.43) and C5S5 (skewness coefficient 0.71) were also skewed to lower yields, but less so than C1S7 and C7S1 (S5 Fig). Hence, there is an association of low median yields and yields skewed towards low yields with a high coefficient of variation within HEs. (Table 4, and S5 Fig). High median yields skewed to high yields are associated with a low coefficient of variation.

**Table 4:** Descriptive statistics of plantain yield across Homologous Events for monocropping.

	C1S7^a^	C4S7^a^	C5S5^a^	C6S6^a^	C7S1^a^
Max	20	25.6	24.74	14.55	15
Mean	7.27	11.78	9.2	11.15	3.95
Median	5.28	11.8	7.88	12.73	3.2
Min	1.2	0.25	1.6	0.85	0.6
Skew	1.12	0.43	0.71	-1.55	1.86
StDev	6.04	7.43	5.27	3.81	3.22
CoefVar	83.17	63.13	57.23	34.19	81.66

^a^ Homologous Events. C indicates climate, whereas S specifies soils, Combination of CS designates each homologous events considering both environmental factors

As in the case of intercropping, the quincunx system yields were generally greater than those with square planting (S6 Fig). However, in all cases one system was dominant within a particular HE (Table 3). Hence, it is not possible to affirm which system was best in a given HE. Similarly, each of the individual HEs was associated with particular varieties, as indicated by the Fisher test, invalidating comparison of varieties within or across HE (Table 3). However, the data from C1S7 suggests that in this HE, square planting with Harton generally produced less than quincunx and Dominico-harton. However, this conclusion is not definitive as one plot with both square and Harton produced more than 20 ton ha^-1^ yr^-1^ (S6 and S7 Figs).

## Discussion

The forest methodology indicated the importance of individual factors as yield determinants, but did not provide useful insights into how combinations of factors affect yield. However, the difference in importance of distinct factors in the monocropping and the intercropping system indicated that combinations of factors are indeed important. This can be better understood by a simple example. In a field with a sandy soil, a gentle slope and a relatively uniform rainfall distribution, drainage is unlikely to be positively associated with yield, whereas on a heavy soil, on flat terrain with a high water table, drainage is likely to be extremely importance. This highlights the importance of looking at combinations of NCFs, rather than individual analysis of their effects. These combinations correspond to HEs.

Several distinct HEs were identified based on NCFs initially identified as potentially important by experienced agronomists, extension agents and agricultural scientists. Each of these HEs encompass circumstances that are sufficiently similar to be considered as recommendation domains, in which the response to management practices will be similar.

The HEs were characterized with climatic data obtained from publicly available databases and soil information collected by farmers and extension agents *in situ*. Factorial analysis was then used to reduce the number of variables. The procedures used, including conditional forest, depended on a large dataset to quantify the impact of distinct CFs in particularly, well defined recommendation domains. It is difficult to quantify these factors with a small number observations.

In spite of more than 900 observations, the multi-stage analytical process produced a very limited number of HEs with a large range of variation within each one. Experienced agronomists and agricultural scientist considered the range of conditions within the HEs too large for the HEs to be considered as valid recommendation domains. A pragmatic subjective assessment of what agronomists considered to be a reasonable number of recommendation domains or HEs, to cover the range of conditions over which the data was collected, was used to set the maximum number of clusters. Within this framework the data was reanalyzed to give seven climate and seven soil clusters.

Expert opinion on its own is appropriate for defining HEs in those crops which have been well researched, but in the case of less well researched crops their performance over a range of conditions can guide the delimitation of HEs [3,27,28]. In sugarcane, (*Saccharum officinarum*) and coffee (*Coffea arabica)*, a wealth of knowledge about the crops, generated through formal experiments and informal experiences, equipped researchers and growers with information to define the NCFs (e.g., climate), CFs (e.g., specific management approaches), likely to influence production [3,42,70]. For Andean blackberry (*Rubus glaucus*) and lulo (*Solanum quitoense*) there was less formal knowledge regarding the likely influence of specific environmental conditions on crop responses, nor was there as much detailed knowledge regarding the functional relationships between processes that may be linked to crop performance. In those crops, with limited formal knowledge of the relevance of the variables that describe non-controllable factors, a mix of expert guidance and automated processing appear to be an effective approach that takes advantages of the synergies between expert knowledge and standardized statistical procedures [28].

A key point in validating the approach of using HEs or recommendation domains is their ability to improve the models used to explain productivity. The lowest Akaike information criterion in the mixed models was obtained by including HEs as a random effect. This indicates that HEs are explaining more of the variation than would be expected by chance. This in turn confirms the viability of the concept of HEs as a means of reducing the stochastic variance inherent in data collected by farmers over a wide range of conditions. Furthermore, it corroborates the methodology based on expert opinion coupled with data mining techniques. As HEs explain a significant part of the variance, their inclusion in the analysis should help reveal how CFs influence productivity both within and across recommendation domains.

In both datasets with monocropping and intercropping, the HEs and specific CFs were frequently confounded. Confounded dataset are likely to be a problem with data from commercial farms, especially if most farmers use similar technology. This may well be the case when most farmers ascribed to a particular HE are from the same geographical region: farmers in the same geographical area frequently use similar practices and plant the same varieties. However, if cross sectional data is obtained with production units from geographically distinct zones, but with similar HEs, there is a greater probability that distinct management practices will appear in the datasets for a given HE, and the likelihood of management practices being confounded with HEs is reduced. Thus, in Andean blackberry the productivity of two geographically distinct regions differed markedly even though their HEs were similar. Assuming that the differences were not due to distinct growing conditions or HEs, the differences were attributed to management [27]. Thus, strategies that obtain datasets from production units with similar NCFs, but from distinct locations, or datasets with ample variation in the same HEs even though geographically concentrated, are advantageous.

We suspect that the large variation in yield within HEs in our datasets indicate that there were indeed large variations in management. Furthermore, the highly skewed yield distribution with a preponderance of very low yield plots under similar NCFs suggests that CFs or management of most plots is sub-optimal for yield. Similar results have been obtained with small farmers in the Andes with Andean blackberry and lulo [27,28]. In the absence of detailed data on management practices, such as fertilizer use, and disease, pest and weed control, we used the geographic region as a proxy for general management levels as suggested in the studies of lulo and Andean blackberry [26, 27]. Management skill, estimated by regional proxy, profoundly influenced yield within HEs with a clear advantage in yield for the *TecnCoffee* proxy. This, once again, supports the approach of HEs or recommendation domains to explain variance in yield due to NCFs and hence to detect how CFs affect yield within a given set of conditions.

However, lack of information on critical CFs, such as fertilizer levels and pest, diseases and weed management, made it impossible to link yields to particular management practices in specific HEs other than the quincunx planting pattern, planting density and variety. The apparently large yield increase associated with the quincunx planting pattern in one HE (C1S7, S4 Fig) was also questionable. Experienced agronomists and agricultural scientists’ opinion proposed that the association in intercropped plantain was likely related to the general technological level of farmers who used quincunx (see eq 2) from one area apparently using similar technology to obtain higher yields than average value. Similarly, the large apparent effects of variety must be treated with care as particular varieties were associated with specific HEs.

The predominance of a single variety in each of the HEs raises the question as to whether the differences in yield ascribed to HEs are due to the variety or the HE. We suggest that farmers generally choose a variety suitable for their particular conditions. The concentration of varieties in specific HEs, across geographical regions, suggests that the definition of HEs is in accordance with farmers’ appreciation of their growing conditions and that they have chosen varieties that fit with their NCFs. Furthermore, it becomes inappropriate to determine what is the cause of the yield difference between the HEs. The differences are presumably due to the combination of the characteristics of the HE and the variety that farmers perceive to be best suited to that HE.

In the intercropping system, across all technological levels and HEs there was a tendency for yield per plant to decrease as plant density increased. This agrees with the general observation in controlled experiments that as plant density increases, individual plant yield decreases. This, in turn, demonstrates that farm data put into the context of HEs can reveal the response of the crop to CFs, such as plant density.

With the tools and data available, HEs or NCFs were clearly defined and furthermore CFs were shown to be important in determining yield. For management practices such as plant density, variety, and planting pattern in intercropping, we only obtained tenuous relationships with yield in spite of having more than one thousand events to analyze and large variations in yield within HEs. We suggest that sufficient reliable data existed to define the HEs, whereas for CFs, even with a large number of events, there was insufficient data on management practices such as fertilizer use and pest management.

Thus, climate data from WorldClim and soil and terrain information evaluated *in situ* with the specially designed methodology of RASTA were sufficient to characterize recommendation domains and effectively define HEs. On the other hand, management data, obtained largely from interviews with growers, were incomplete and insufficient to characterize the individual CFs. It was not possible to obtain data on fertilizer use and disease, pest and weed control. Most of the growers had no formal farm records to help them recall how they managed their crops. Thus, lack of farm records on crop management practices severely limited the ability to determine which practices are appropriate in particular HEs or recommendation domains. In addition, the farmers’ data based solely on memory contained errors. These included obvious errors, such as unreal planting densities and confusion of units of yield. These errors were corrected wherever possible. Nevertheless, the lack of farm records is a major obstacle to effective use of farmer information and knowledge.

Howland et al. [73], in a parallel study, found few small fruit farmers kept good farm records. Many of them saw the advantage of keeping records, but they had neither the skills required nor the tools needed to keep records. Currently few growers have access to internet or computer skills, and hence they were not able to use the platform developed as part of the project to maintain adequate records and continually monitor their crop performance. Furthermore, the lack of access to modern Information and Communication Technologies (ICTs) and the generally low levels of education in rural areas minimize the ability of farmers to access the results of analysis presented here [73,74]. Nevertheless, growers enrolled in farmers field schools showed interest in sharing information and were capable of interpreting information and using it to make informed decisions when it was presented to them in a format they could understand [73,75,76]. This suggests that the use of farmer generated information as proposed in this paper will only be an effective tool if farmers: (a) improve their capacity to keep better records; (b) are provided with the means to keep records; and (c) via group activities such as farmers field schools, receive processed information in a format that they can use to make better decisions.

In Australia and Chile, CropCheck has been developed as a system which growers use to keep records on management practices that influence yield [6]. Experienced extension agents define those practices that are likely to influence yield and design protocols for recording them. Growers then record whether they apply these practices or not. The data from many farmers are analyzed and associations between yield and distinct practices quantified. CropCheck has been used in relatively uniform conditions, unlike those in the mountainous regions in which we work. CropCheck looks at the genetic by management interactions, but does not include their interaction with the environment, the Genetic by Management by Environment interactions (G x M x E). Farmers using the CropCheck system obtain higher yields [6,77]. We suggest that the deficiency in data on CFs we encountered could be alleviated by adopting systems like CropCheck. Such a system, when coupled with the approach of NCFs, would be suitable for situations with greater heterogeneity than those for which CropCheck was developed.

Although the lack of information on CFs in our study means that it is not possible to directly deduce optimal practices for a particular HE, an interesting option emerged. The generally low yields in the HEs, C1S7 and C7S1, coupled with the occasional high yields indicated that most farmers were not using cultural practices that exploited the yield potential of the HE. However, it was possible to identify those farmers that were obtaining high yields. Hence, if the farmers with low yields were able to visit and exchange information with the farmers with high yield, it is likely that the low yield producers of today could become the high yield farmers of tomorrow. Small-scale Colombian fruit farmers are indeed willing to share information [73,75,76]. Hence, simply identifying those farmers who produce high yields in a particular recommendation domain opens the way to increasing the yields of all growers, even though specific practices are not identified.

## Conclusions

We revisited the idea of recommendation domains from a bottom-up perspective and developed methodologies for determining Homologous Events (HEs) with similar non-controllable factors. Data from sources such as WorldClim, and farmers’ observations such as RASTA for soils, were analyzed. Successful data mining techniques to define HEs were only possible when guided by expert opinion. Expert opinion was strengthened and supported by rigorous analytical methods. The addition of HEs to mixed models improved their capacity to explain yield variation, confirming the original hypothesis that the recommendation domain concept can be applied effectively at the micro-level. Furthermore, the use of HEs, which bring together combinations of NCFs, was shown to be more effective than looking at the effects of individual factors and their interactions. The lack of detailed information on management practices limited the ability to define which individual practices were beneficial in a particular recommendation domain. However, the use of proxies for general management ability clearly demonstrated that differences in management are related to yield variation within recommendation domains and that well managed farms can be identified. This opens the way for using HEs to identify good farmers in a particular recommendation domain: these farmers can then share their information with other less productive growers who may improve their productivity. In spite of the limited data on management practices, we demonstrated that when data is available it is possible to associate increased productivity with particular management practices within an HE. Hence, lack of good farm records is currently a major limitation to the approach, but systems such as CropCheck exist in other countries and could be adopted. The combination of systems such as CropCheck, which only looks at CFs, with the concepts of HEs which incorporate NCFs would appear to be extremely powerful.

## Supporting Information

S1 Fig Histogram displaying yield data distribution of plantain. (a) monocropping, (b) intercropping.
(TIFF)

S2 Fig Clusters of climate defined by the K-means algorithm. (TIFF)

S3 Fig Clusters of soils defined by the K-means algorithm. (TIFF)

S4 Fig Yield and planting pattern in several HEs for intercropping (kg plant^-1^ yr^-1^). (TIFF)

S5 Fig Histogram displaying yield distribution across HEs for monocropping (ton ha^-1^ yr^-1^). (TIFF)

S6 Fig Yield and planting pattern in several HEs for monocroping (ton ha^-1^ yr^-1^). (TIFF)

S7 Fig Yield and variety in several HEs for monocroping (ton ha^-1^ yr^-1^). (TIFF)

## References

[1] Harlan J. Crops and Man American Society of Agronomy. Madison, Wisconsin 1975.

[2] Delmotte S, Tittonell P, Mouret J-C, Hammond R, Lopez-Ridaura S. On farm assessment of rice yield variability and productivity gaps between organic and conventional cropping systems under Mediterranean climate. Eur J Agron. Elsevier B.V.; 2011;35: 223–236. 10.1016/j.eja.2011.06.006

[3] Cock J, Oberthür T, Isaacs C, Läderach PR, Palma A, Carbonell J, et al Crop Management Based on Field Observations: case studies in sugarcane and coffee. Agric Syst. 2011;104: 755–769. 10.1016/j.agsy.2011.07.001

[4] Parsa S, Ccanto R, Olivera E, Scurrah M, Alcázar J, Rosenheim J a. Explaining Andean potato weevils in relation to local and landscape features: a facilitated ecoinformatics approach. PLoS One. 2012;7: e36533 10.1371/journal.pone.0036533 22693551 PMC3365062

[5] Steinmann KP, Zhang M, Grant JA. Does use of Pesticides Known to Harm Natural Enemies of Spider Mites (Acari: Tetranychidae) Result in Increased Number of Miticide Applications? An Examination of California Walnut Orchards. Journal of Economic Entomology. 2011 pp. 1496–1501. 10.1603/EC11168 22066177

[6] Lacy J. Cropcheck: Farmer benchmarking participatory model to improve productivity. Agric Syst. 2011;104: 562–571. Available: http://www.sciencedirect.com/science/article/pii/S0308521X1100059X

[7] Evans LT, Fischer RA. Yield Potential: Its Definition, Measurement, and Significance. Crop Sci. 1999;39: 1544–1551.

[8] Chambers R, Ghildyal B. Agricultural Research for Resource Poor Farmers -The Farmer First and Last Model. Agric Adm. 1985; 1–30.

[9] Chambers R, Pacey A, Thrupp L. Farmer First: Farmer Innovation and Agricultural Research Intermed Technol Publ London. 1989

[10] Pretty J. Farmers’ Extension Practice and Technology Adaptation: Agricultural Revolution in 17-19th Century Britain. Agric Human Values. 1991;8: 132–148.

[11] Thompson J, Scoones I. Challenging The Populist Perspective: Rural People’s knowledge. Agric Res Ext Pract Agric Hum Values. 1994;11: 58–76.

[12] Marsh SP, Pannell D. Agricultural extension policy in Australia: the good, the bad and the misguided. Aust J Agric Resour Econ. 2000;44: 605–627.

[13] Russell DB, Ison RL. The Research Development Relationship in Rural Communities: An opportunity for contextual science In: Agricultural and Extension and Rural Development: Breaking out of traditions (Eds): Cambridge University Press pp. 10–29. 2000;

[14] Altieri MA. The science of natural resource management for poor farmers in marginal environments. Agric Ecosyst Environ. 2002;171: 1–24.

[15] Hall A. Capacity development for agricultural biotechnology in developing countries:an innovation systems view of what it is and how to develop it. J Int Dev. 2005;17: 611–630.

[16] Van Asten PJA, Kaaria S, Fermont AM, Delve RJ. Challenges and Lessons When Using Farmer Knowledge in Agricultural Research and Development Projects in Africa. Exp Agric. 2008;45: 1 10.1017/S0014479708006984

[17] Landini F. Problemas de la extensión rural en América Latina. Perfiles Latinoam. 2016;24: 47–68. 10.18504/pl2447-005-2016

[18] Rist G. The History of Development: from Western Origins to Global Faith. New Edition Zed Books London 1997;

[19] Cukier K, Mayer-Schönberger V. The Rise of Big Data: How It’s Changing the Way We Think About the World. Foreign Aff. 2013;92: 1–7.

[20] Zee F. Modeling of Plant-Based Life Support Processes Using Artificial Neural Networks [Internet]. Advanced Life Support and Advanced Environmental Monitoring and Control Workshop Houston, Texas, USA. 1997. Available: http://hdl.handle.net/2014/22797

[21] Bänziger M, Setimela PS, Hodson D, Vivek B. Breeding for improved abiotic stress tolerance in maize adapted to southern Africa. Agric Water Manag. 2006;80: 212–224. 10.1016/j.agwat.2005.07.014

[22] Braun H-J, Rajaram S., Van Ginkel M. CIMMYT’s approach to breeding for wide adaptation. Euphytica. 1996;92: 175–183.

[23] Cock J. Stability of Performance of Cassava Genotypes In: Hershey C.H. (Eds.): Proceeding Workshop Cassava Breeding. A Multidisciplinary Review. Los Banos, Philippines; 1985 pp. 177–206.

[24] Gauch HG, Zobel RW. Identifying mega-environments and targeting genotypes. Crop Sci. 1997;37: 311–326.

[25] Harrington L, Tripp R. Recommendation Domains: A Framework for On-Farm Research. CIMMYT Econ Progr Work Pap. 1984;2: 27.

[26] Gitterle T, Martinez W, Marimon F, Salazar M, Faillace J, Suarez A, et al Commercial field performance as a measure of genetic improvement in the Pacific White Shrimp Penaeus (Litopenaeus) vannamei International Symposium of Genetics in Aquaculture. Bangkok, Thailand 2009.

[27] Jiménez D, Cock J, Satizábal HF, Barreto S MA, Pérez-Uribe A, Jarvis A, et al Analysis of Andean blackberry (Rubus glaucus) production models obtained by means of artificial neural networks exploiting information collected by small-scale growers in Colombia and publicly available meteorological data. Comput Electron Agric. 2009;69: 198–208.

[28] Jiménez D, Cock J, Jarvis A, Garcia J, Satizábal HF, Van Damme P, et al Interpretation of commercial production information: A case study of lulo (Solanum quitoense), an under-researched Andean fruit. Agric Syst. 2011;104: 258–270.

[29] Satizábal H, Barreto-Sanz M, Jiménez D, Pérez-Uribe A, Cock J. Technologies and Innovations for Development [Internet]. Bolay J-C, Schmid M, Tejada G, Hazboun E, editors. Paris: Springer Paris; 2012.

[30] Agrawal RC, Heady O. Operations Research Methods for Agricultural Decisions [Internet]. Iowa State University Press; 1972 Available: http://books.google.com.co/books?id=MisRRAAACAAJ

[31] NRC. Precision Agriculture in the 21st Century: Geospatial and Information Technologies in Crop Management. Committee on Assessing Crop Yield: Site-Specific Farming, Information Systems, and Research Opportunities. National Academy Press, Washington D.C. pp. 7. 1997

[32] Cassman K. Ecological intensification of cereal production systems: Yield potential, soil quality, and precision agriculture. Proc Natl Acad Sci United States Am. 1999;96: 5952–5959. 10.1073/pnas.96.11.5952 PMC34211 10339523

[33] Spaans E, Estrada L.. Sense and nonsense of satellite navigaton for precision agriculture in the tropics. Eur J Navig. 2004;2: 71–76.

[34] Barton D. Making Advanced Analytics Work For You. Harv Bus Rev. 2012; 23074867

[35] Mayer-Schonberger V, Cukier K.). Big Data: A Revolution That Will Transform How We Live, Work and Think. 2013.

[36] Jiménez D, Pérez-Uribe A, Satizabal HF, Barreto M, Van Damme P, Tomassini M. A survey of artificial neural network-based. Modeling in agroecology In: Prasad B, editor. Soft Computing Applications in Industry. Berlin, Heidelberg: Springer Berlin Heidelberg; 2008 pp. 247–26.

[37] Álvarez E, Ceballos G, Gañan L, Rodríguez D, González S, Pantoja A. Producción de material de “siembra” limpio en el manejo de las enfermedades limitantes del plátano [Internet] Vasa. Cali, Colombia; 2013 Available: http://www.fao.org/docrep/019/as090s/as090s.pdf

[38] Alvarez DM, Estrada M, Cock JH, Cock JH. RASTA (Rapid Soil and Terrain Assessment) Facultad De Ciencias Agropecuarias. Universidad Nacional De Colombia 2004.

[39] Hijmans RJ, Cameron SE, Parra JL, Jones PG, Jarvis A. Very high resolution interpolated climate surfaces for global land areas. Int J Climatol. 2005;25: 1965–1978.

[40] Strobl C, Boulesteix A-L, Zeileis A, Hothorn T. Bias in random forest variable importance measures: illustrations, sources and a solution. BMC Bioinformatics. 2007;8: 25 10.1186/1471-2105-8-25 17254353 PMC1796903

[41] Strobl C, Boulesteix A, Kneib T, Augustin T, Zeileis A. Conditional variable importance for random forests. BMC Bioinformatics. 2008;11: 1–11. 10.1186/1471-2105-9-307 PMC2491635 18620558

[42] Niederhauser N, Oberthür T, Kattnig S, Cock J. Information and its management for differentiation of agricultural products: the example of specialty. Comput Electron Agric. 2008;61: 241–253.

[43] Lin CY, Musta B, Abdullah MH. Geochemical processes, evidence and thermodynamic behavior of dissolved and precipitated carbonate minerals in a modern seawater/freshwater mixing zone of a small tropical island. Appl Geochemistry. Elsevier Ltd; 2013;29: 13–31. 10.1016/j.apgeochem.2012.10.029

[44] Ludovic L, Morineau A, Piron M. Statistique exploratoire multidimensionnelle. 1997.

[45] Pecher C, Tasser E, Walde J, Tappeiner U. Typology of Alpine region using spatial-pattern indicators. Ecol Indic. Elsevier Ltd; 2013;24: 37–47. 10.1016/j.ecolind.2012.05.025

[46] Verfaillie E, Degraer S, Schelfaut K, Willems W, Van Lancker V. A protocol for classifying ecologically relevant marine zones, a statistical approach Estuar Coast Shelf Sci. Elsevier Ltd; 2009;83: 175–185. 10.1016/j.ecss.2009.03.003

[47] Zafiriou P, Mamolos AP, Menexes GC, Siomos AS, Tsatsarelis C a., Kalburtji KL. Analysis of energy flow and greenhouse gas emissions in organic, integrated and conventional cultivation of white asparagus by PCA and HCA: cases in Greece. J Clean Prod. 2012;29–30: 20–27. 10.1016/j.jclepro.2012.01.040

[48] Ben-Hur A, Guyon I. Detecting stable clusters using principal component analysis. Methods Mol Biol. 2003;224: 159–82. 10.1385/1-59259-364-X:159 12710673

[49] Husson F, Josse J, Pages J. Principal component methods—hierarchical clustering—partitional clustering: why would we need to choose for visualizing data? Technical Report of the Applied Mathematics Department (Agrocampus). 2010.

[50] Cane JH. Soils of Ground-Nesting Bees (Hymenoptera : Apoidea): Texture, Moisture, Cell Depth and Climate. J Kansas Entomol Soc. 1991;64: 406–413.

[51] Dinpashoh Y, Fakheri-Fard a, Moghaddam M, Jahanbakhsh S, Mirnia M. Selection of variables for the purpose of regionalization of Iran’s precipitation climate using multivariate methods. J Hydrol. 2004;297: 109–123. 10.1016/j.jhydrol.2004.04.009

[52] Mimmack G, Mason S, Galpin J. Choice of distance matrices in cluster analysis: Defining regions. J Clim. 2001; 2790–2797. Available: http://journals.ametsoc.org/doi/abs/10.1175/1520-0442(2001)014%3C2790%3ACODMIC%3E2.0.CO%3B2

[53] Muñoz-Díaz D, Rodrigo FS. Spatio-temporal patterns of seasonal rainfall in Spain (1912–2000) using cluster and principal component analysis: comparison. Ann Geophys. 2004;22: 1435–1448. 10.5194/angeo-22-1435-2004

[54] Schoof JT, Pryor SC. Downscaling temperature and precipitation: a comparison of regression-based methods and artificial neural networks. Int J Climatol. 2001;21: 773–790. 10.1002/joc.655

[55] Unal Y, Kindap T, Karaca M. Redefining the climate zones of Turkey using cluster analysis. Int J Climatol. 2003;23: 1045–1055. 10.1002/joc.910

[56] García L. JC, Posada-Suárez H, Läderach P. Recommendations for the Regionalizing of Coffee Cultivation in Colombia: A Methodological Proposal Based on Agro-Climatic Indices. PLoS One. 2014;9: e113510 10.1371/journal.pone.0113510 25436456 PMC4250036

[57] Linting M, Meulman JJ, Groenen PJF, van der Koojj AJ. Nonlinear principal components analysis: introduction and application. Psychol Methods. 2007;12: 336–58. 10.1037/1082-989X.12.3.336 17784798

[58] Burghelea CI, Zaharescu DG, Hooda PS, Palanca-Soler A. Predatory aquatic beetles, suitable trace elements bioindicators. J Environ Monit. 2011;13: 1308–15. 10.1039/c1em10016e 21468408

[59] Campos-Herrera R, Gómez-Ros JM, Escuer M, Cuadra L, Barrios L, Gutiérrez C. Diversity, occurrence, and life characteristics of natural entomopathogenic nematode populations from La Rioja (Northern Spain) under different agricultural management and their relationships with soil factors. Soil Biol Biochem. 2008;40: 1474–1484. 10.1016/j.soilbio.2008.01.002

[60] Dossa L, Abdulkadir A, Amadou H, Sangare S, Schlecht E. Exploring the diversity of urban and peri-urban agricultural systems in Sudano-Sahelian West Africa: An attempt towards a regional typology. Landsc Urban Plan. Elsevier B.V.; 2011;102: 197–206. 10.1016/j.landurbplan.2011.04.005

[61] Rouabhi A, Hafsi M, Kebiche M. RTICLE Assessment Of The Relationship Between The Typology And Economic Performance Of Farms: A Case Study For A Rural Area Of Province Setif, Algeria 1. 2012;6: 2259–2268.

[62] SPSS Inc M. PASW Statistics 18 Guide to Data Analysis, 1st edition. PASW Stat 18 Guid to Data Anal 1st Ed PASW Stat 18 Guid to Data Anal 1st Ed. 2010; papers2://publication/uuid/F3EF6A76-6319-4C7E-A1EF-0698F2F3DC67

[63] Robinson GK. That BLUP is a Good Thing: The Estimation of Random Effects. Stat Sci. 1991;6: 15–32.

[64] Rabe-Hesketh S, Skrondal A. Multilevel and Longitudinal Modeling Using Stata, 2nd edition Texas pp.156–160: Stata Press,College Station; 2008.

[65] Deadman P, Gimblett HR. An Application of Neural Net Based Techniques and GIS for Vegetation Management and Restoration AI Applications. 1997 http://www.srnr.arizona.edu/~gimblett/aidead97.html

[66] Verbeke G, Molenberghs G. Linear Mixed Models for Longitudinal Data. Linear Mixed Models in Practice. Springer New York; 1997 pp. 63–153. 10.1007/978-1-4612-2294-1_3

[67] Yan W, Hunt LA, Johnson P, Stewart G, Lu X. On-Farm Strip Trials vs. Replicated Performance Trials for Cultivar. Crop Sci. 2002;42: 385–392.

[68] Carbonell GJ, Amaya EA, Ortiz BV, Torres JS, Quintero R, Isaacs C. Zonificación agroecológica para el cultivo de caña de azúcar en el valle del río Cauca. (Agro-ecological zoning for the sugarcane crop in the Cauca River Valley.) Tercera aproximación Tech Ser CENICAÑA no 29 Cali, Colomb.

[69] Liu M, Samal A. A fuzzy clustering approach to delineate agroecozones. Ecol Modell. 2002;149: 215–228.

[70] Isaacs CH, Carbonell JA, Amaya A, Torres JS, Victoria JI, Quintero R, et al. Site Specific Agriculture And Productivity In The Colombian Sugar Industry. In: Proceedings of the 26th congress International Society of Sugar Cane Technologists (ISSCT),. Durban, South Africa; 2007.

[71] Zuur AF, Ieno EN, Walker NJ, Saveliev AA, Smith GM, Ebooks Corporation. Mixed Effects Models and Extensions in Ecology with R [Internet] Statistics for Biology and Health. 2009 10.1007/978-0-387-87458-6

[72] McGilchrist CA. Estimation in Generalized Mixed Models. J R Stat Soc. 1994;56: 61–69.

[73] Howland F, Muñoz LA, Staiger-Rivas S, Cock J, Alvarez S. Data sharing and use of ICTs in agriculture : working with small farmer groups in Colombia. Knowl Manag Dev J. 2015;11: 44–63. Available: http://journal.km4dev.org/

[74] Chaparro F, Cock J. Estrategias para fomentar la innovación en el sector agropecuario como locomotora del desarrollo rural en Colombia Misión de Ciencia, Educación y Desarollo—Balance 20 años después. Bogotá; 2015: 121–131.

[75] ASOHOFRUCOL. Boletín Informativo AESCE [Internet] Boletín Informativo AESCE Diciembre 2015. Bogotá; 2015 Available: http://www.frutisitio.com/index.php?option=com_content&view=article&id=104&Itemid=545

[76] ASOHOFRUCOL. Boletín Informativo AESCE [Internet]. Boletín Informativo AESCE Agosto 2015. Bogotá; 2015 Available: http://www.frutisitio.com/index.php?option=com_jdownloads&task=download.send&id=11&catid=2&m=0&Itemid=526

[77] Araya F, Acevedo R, Cabello MC, Jaramillo C, Gonzalez I, Toro M. CropCheck Chile:Sistema de Extension para el Sector AgroAlimentario [Internet]. 2nd ed. Tobar. Patricia, editor. Santiago de Chile: Fundación Chile en el Programa Cropcheck; 2010. Available: http://www.gtt.cl/archivos_interes/06.sistema_de_extension_sector_agroalimentario.pdf

