# Supplementary figures and images for: From Observation to Information: Data-Driven Understanding of on Farm Yield Variation
Source: PLoS One. 2016 Mar 1;11(3):e0150015. doi: 10.1371/journal.pone.0150015 (PMC4773236; doi:10.1371/journal.pone.0150015)

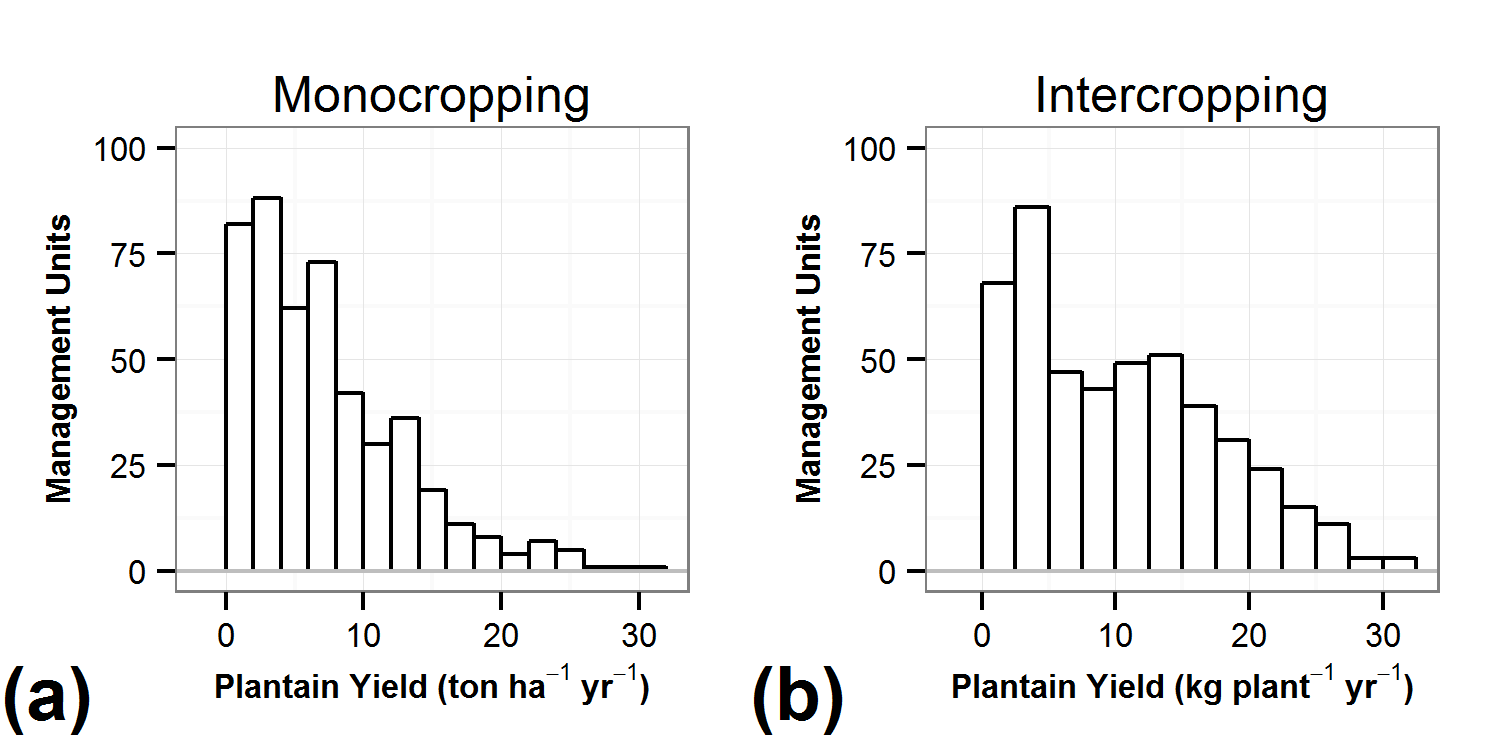

Supplement: S1 Fig — (a) monocropping, (b) intercropping. (TIFF) [file pone.0150015.s001.tiff]

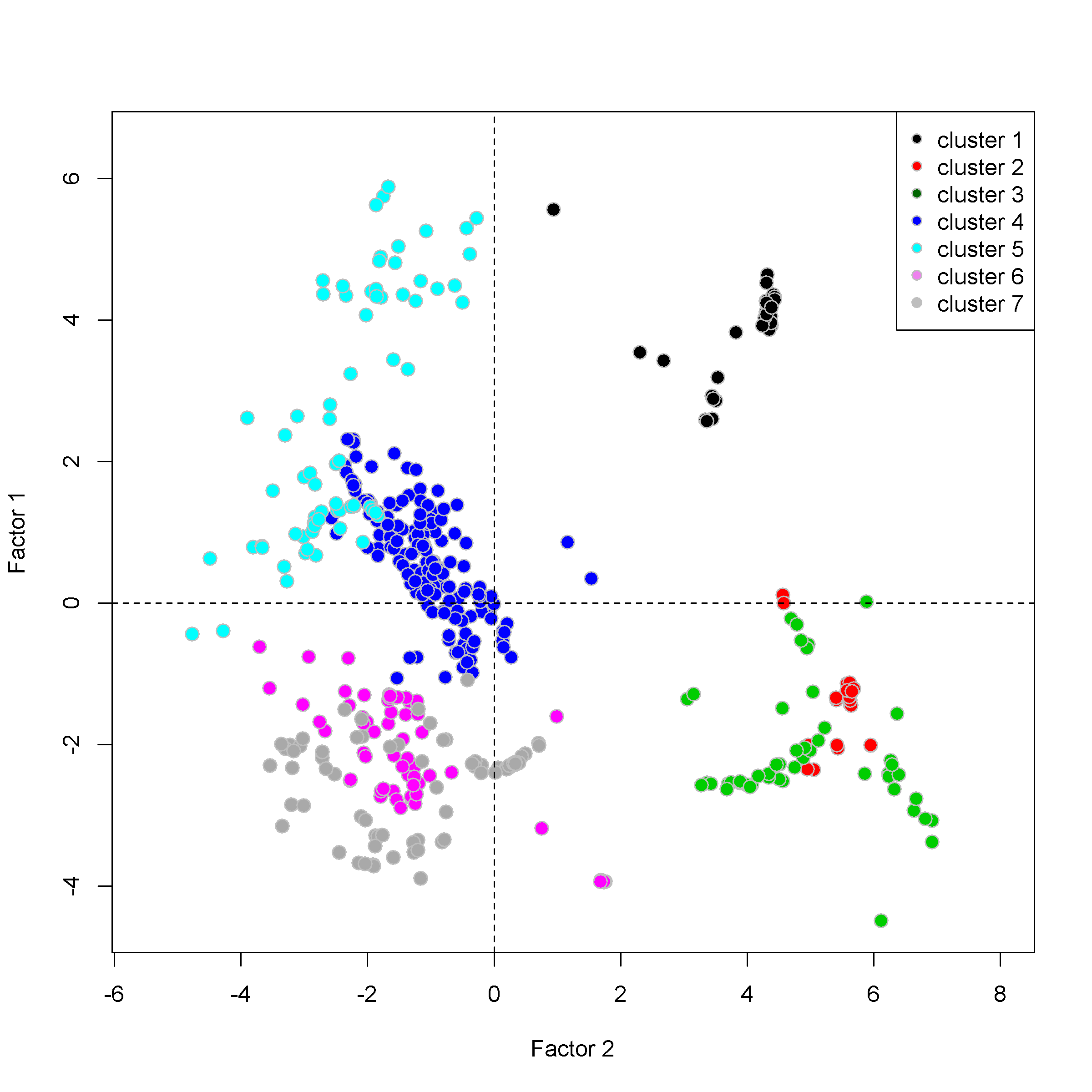

Supplement: S2 Fig — (TIFF) [file pone.0150015.s002.tiff]

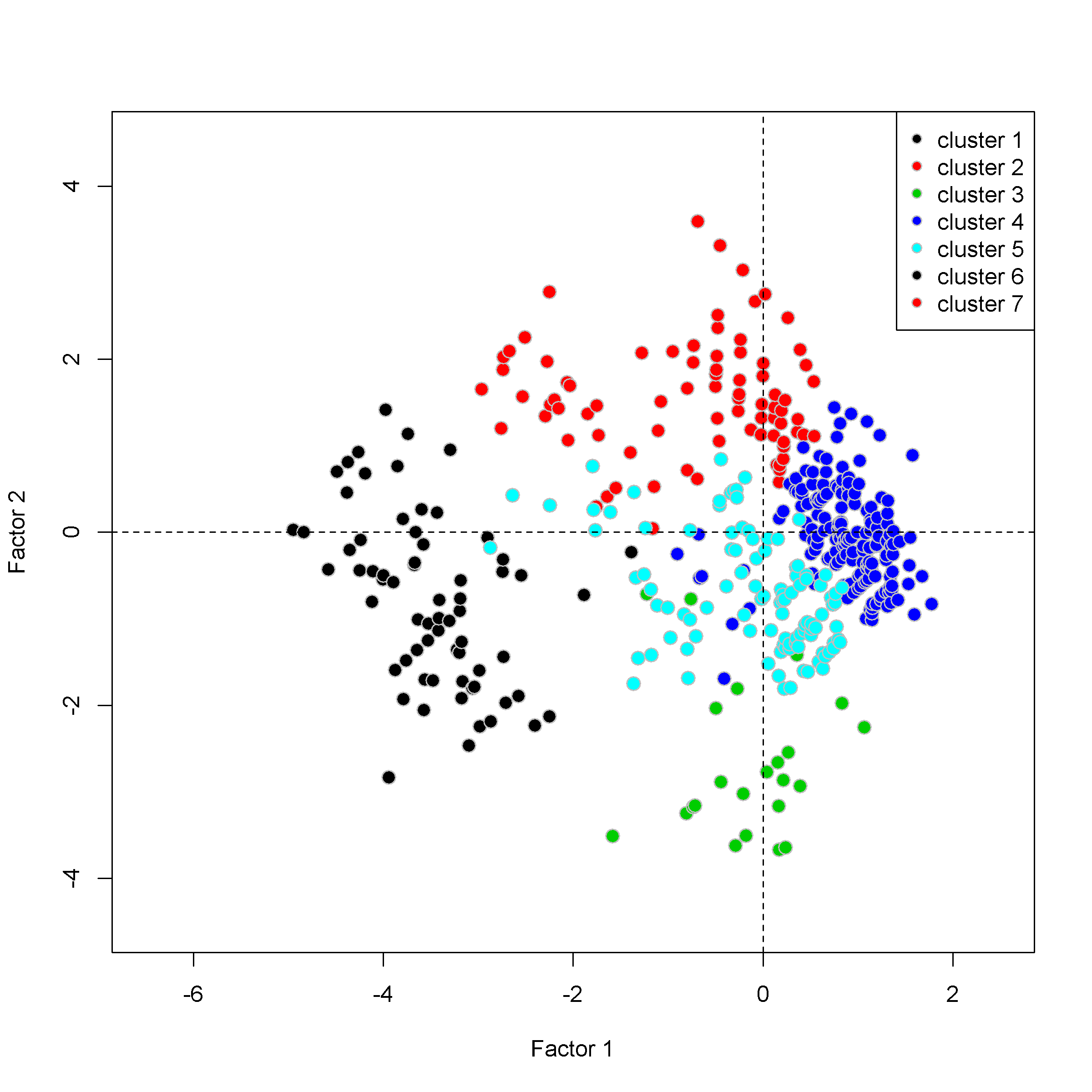

Supplement: S3 Fig — (TIFF) [file pone.0150015.s003.tiff]

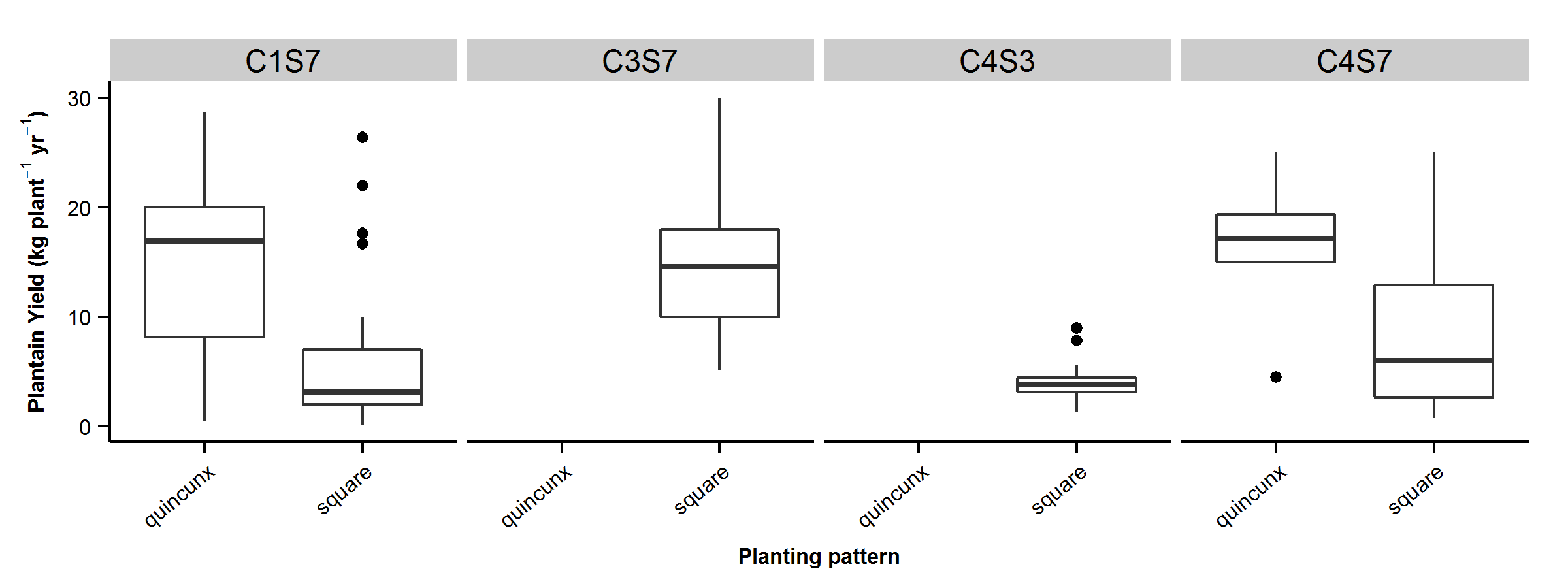

Supplement: S4 Fig — (TIFF) [file pone.0150015.s004.tiff]

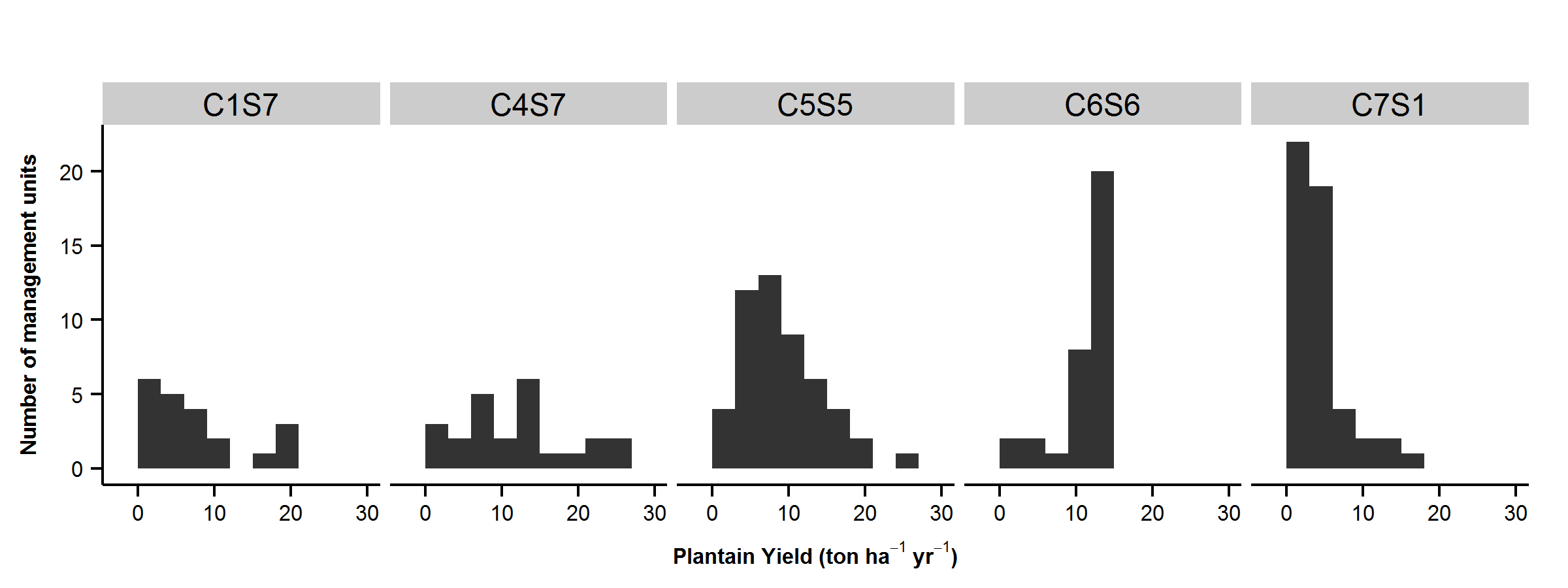

Supplement: S5 Fig — (TIFF) [file pone.0150015.s005.tiff]

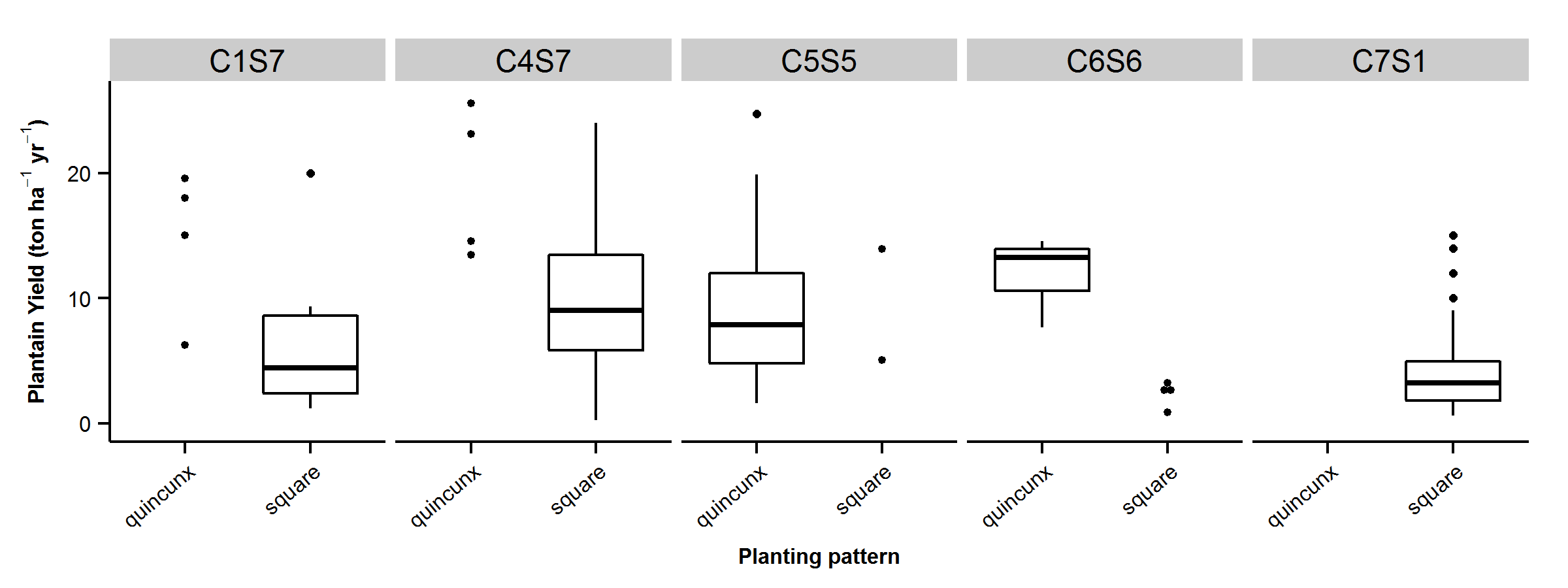

Supplement: S6 Fig — (TIFF) [file pone.0150015.s006.tiff]

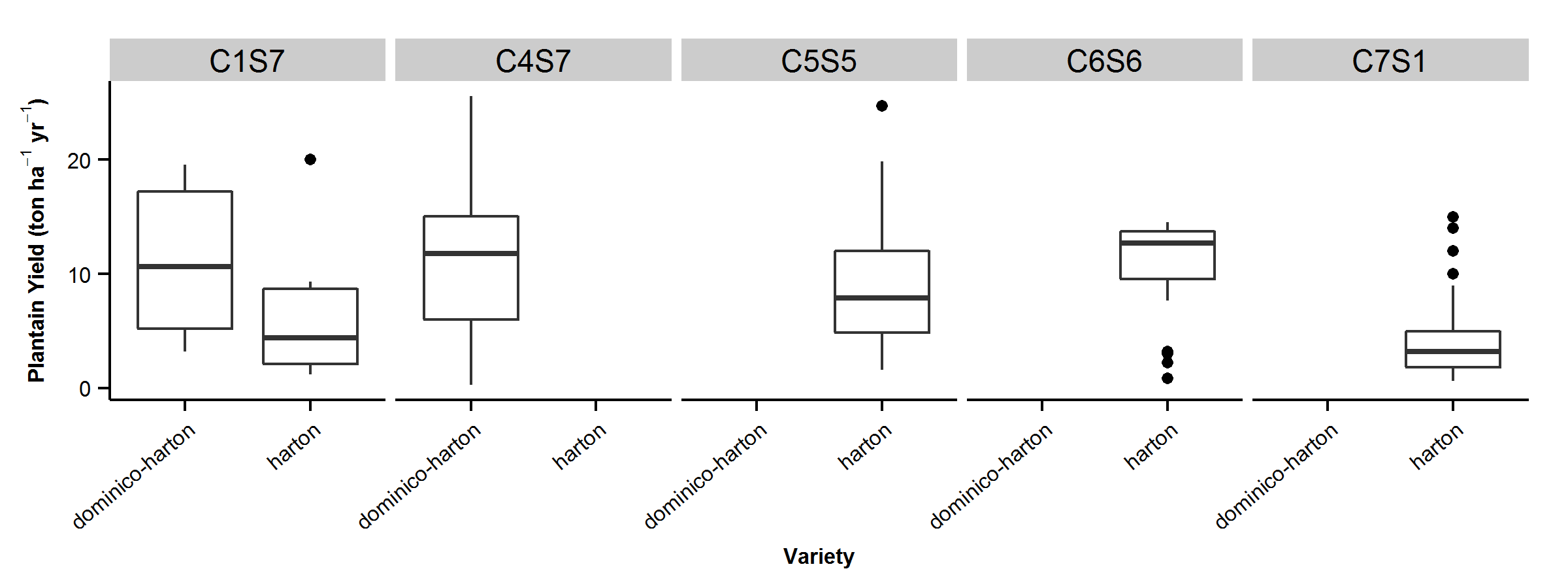

Supplement: S7 Fig — (TIFF) [file pone.0150015.s007.tiff]
